# White Matter Abnormalities in Two Different Subtypes of Amnestic Mild Cognitive Impairment

**DOI:** 10.1371/journal.pone.0170185

**Published:** 2017-01-20

**Authors:** Jianghong Liu, Peipeng Liang, Linlin Yin, Ni Shu, Tengda Zhao, Yi Xing, Fangyu Li, Zhilian Zhao, Kuncheng Li, Ying Han

**Affiliations:** 1 Department of Neurology, Xuan Wu Hospital, Capital Medical University, Beijing, China; 2 Department of Radiology, Xuan Wu Hospital, Capital Medical University, Beijing, China; 3 Beijing Key Lab of MRI and Brain Informatics, Beijing, China; 4 Department of Pharmacology, Xuan Wu Hospital, Capital Medical University, Beijing Geriatric Medical Research Center, Key Laboratory for Neurodegenerative Disease of Ministry of Education, Beijing, China; 5 State Key Laboratory of Cognitive Neuroscience and Learning, Beijing Normal University, Beijing, China; 6 Center of Alzheimer’s Disease, Beijing Institute for Brain Disorders, Beijing, China; Brainnetome Center & The National Laboratory of Pattern Recognition, CHINA

## Abstract

White matter (WM) degeneration has been found during the course of cognitive decline in both Alzheimer's disease (AD) and amnestic mild cognitive impairment (aMCI), however, it is unclear whether there are different WM microstructural abnormalities between two subtypes of aMCI, including single domain aMCI (aMCI-s) and multiple domain aMCI (aMCI-m). Thirty-two patients of aMCI single-domain (aMCI-s), twenty-three patients of aMCI multiple-domain (aMCI-m) and twenty-three healthy normal controls (NC) participated in this study. Neuropsychological measures and diffusion tensor imaging (DTI) data were acquired from each subject and tract-based spatial statistics (TBSS) was implemented. It was found that both aMCI groups showed significantly reduced fractional anisotropy (FA) in the right superior longitudinal fasciculus (SLF) than NC. It was also identified that, as compared to aMCI-m, aMCI-s showed significantly decreased FA in the left SLF, left uncinate fasciculus (UF) and left inferior longitudinal fasciculus (ILF), while significantly increased FA in the left anterior thalamic radiation (ATR). The correlation analysis showed that FA values in the regions with group difference were significantly correlated with cognitive functions as measured by Boston naming test and trail making test. These results suggested that the variations of aMCI may be differentiated by FA indexes and DTI may help to understand why specific signs and symptoms occur in patients.

## Introduction

Alzheimer’s disease (AD) is a common dementia in elderly populations, and amnestic mild cognitive impairment (aMCI) often represents a transitional stage between normal aging and early dementia [1,2]. Patients with aMCI are at higher risks of evolving toward AD (approximately 10%-15% per year), up to 80% of aMCI individuals would progress to dementia after 6 years [3]. There are two types of aMCI: single-domain of aMCI (aMCI-s) and multiple-domain of aMCI (aMCI-m). aMCI-s have isolated memory impairment, whereas aMCI-m have impairments in multiple cognitive domains including memory, language, executive functions, visuospatial skills, etc. [4]. It is necessary to differential diagnose of aMCI-s and aMCI-m based on objective imaging characteristics not only for the in-depth understanding of degenerative neural changes of aMCI but also for the different treatment and judgment of conversion for the two subtypes.

AD was widely reported to have the neurodegenerative effects on cerebral white matter (WM) microstructure [5,6]. Diffusion tensor imaging (DTI) has been demonstrated to be a useful tool for the early detection of AD as WM changes have been detected in early AD or prodromal AD stages which is known as MCI stages [7]. There are many studies in AD and MCI by DTI, and the differences of fractional anisotropy (FA) and mean diffusivity (MD) were observed in many cortical regions [8–13]. Furthermore, it was also found that WM abnormalities in patients were associated with various cognitive dysfunctions [14,15]. However, less study have focused on patients with aMCI by DTI [16–18] and the findings are heterogeneous. In particular, the WM changes of the two subtypes of aMCI, i.e., aMCI-s and aMCI-m, were not studied so far.

The main goal of this study is to investigate the difference of diffusion indices between aMCI-s and aMCI-m by using tract-based spatial statistics (TBSS), which could improve the sensitivity, objectivity and interpretability of diffusion imaging results at group level [19]. Given aMCI is a kind of disconnection syndrome and significant difference between aMCI-s and aMCI-m, it is hypothesized that both aMCI-s and aMCI-m may have significant alterations in DTI metrics than normal controls (NC), and some DTI indexes may differentiate between aMCI-s and aMCI-m.

## Subjects and Methods

### Subjects

Thirty-two aMCI-s (70.75±6.40 years old; 15 females) and 23 aMCI-m (70.91±8.07 years old; 10 females) patients were screened from the Department of Neurology of Xuanwu Hospital, Capital Medical University from January 2011 to March 2015. Twenty-three NC (64.61±9.11 years old; 13 females) were recruited from local residents. Written informed consents were obtained from all participants or their relatives before the MRI scan. This study was approved by the Institutional Review Board of Xuanwu Hospital, Capital Medical University.

The patients with aMCI were diagnosed according to Petersen’s criteria [4] and National Institute on Aging- Alzheimer’s Association criteria for MCI due to AD [20] as following: (a) memory complaint; (b) objective memory impairment; (c) near-normal performance on general cognition and preserved daily life activities (as measured by Activity of Daily Living Scale (ADL)); (d) Clinical Dementia Rating (CDR) score of 0.5; (e) failure to meet the criteria for dementia according to the Diagnostic and Statistical Manual of Mental Disorders, fourth edition, revised (DSM-IV) [21]; (f) hippocampal atrophy observed (as measured by the Medial Temporal lobe Atrophy scale (MTA-scale)) and (h) the Han nationality, right-handed (the Edinburgh handedness scale score > 40 points). The diagnosis of aMCI-s and aMCI-m were fulfilled according to Petersen’s diagnostic criteria [4].

Participants of NC were cognitively normal and had a CDR of 0. Subjects were excluded if they met the following clinical characteristics: (a) those who have a clear history of stroke (Hachinski Ischemic Scale score (HIS)> 7 points); (b) severe depression (Hamilton Depression Rating Scale score (HAMD) > 24 points); (c) cognitive impairment caused by traumatic brain injury; (d) other nervous system diseases, which could cause cognitive impairment; (e) systemic diseases, which could cause cognitive impairment; (f) a history of psychosis or congenital mental growth retardation; and (g) those who cannot corporate with neuropsychological tests or have any contraindication to Magnetic Resonance Imaging (MRI).

All participants underwent a series of neurological tests and a battery of neuropsychological assessments by two experienced neurologists (JL and YH), including mini-mental state examination (MMSE) [22], Montreal cognitive assessment (MoCA) [23,24], Auditory Verbal Learning Test (AVLT of Chinese version; short delay free recall) [25], Boston naming test (BNT) used by Cheung RW et al [26], trail making test (TMT) [27], clock drawing test (CDT; 3-point) [28] and CDR [29]. Specifically, TMT is a neuropsychological test of visual attention and task switching [27]. It consists of two parts in which the subject is instructed to connect a set of 25 dots as quickly as possible while still maintaining accuracy. There are two parts to the test: in the first part, the targets are all numbers (1, 2, 3, etc.) and the test taker needs to connect them in sequential order; in the second part, the subject alternates between numbers and letters (1, A, 2, B, etc.). The time taken to complete the test is used to be the primary performance metric. We then used 3-point CDT to test the visuospatial skill, TMT to evaluate the executive function, BNT to assess the language skill, and AVLT (short delay free recall) to measure the memory function. In addition, the prevalence of vascular factors such as hypertension, hypercholesterolemia, and heart attack, did not differ among the groups. All subjects had no history of a psychiatric or neuropsychological disease.

### MRI Acquisition

MRI data acquisition was performed on a 3-Tesla scanner (Siemens Medical Solutions, Erlangen, Germany). MRI data were collected the day after the subjects finished the cognitive testing. Sedation was forbidden during the MRI scanning. Foam padding and headphones were used to limit head motion and reduce scanner noise. The 3D T1-weighted anatomical image was acquired with a magnetization-prepared rapid gradient echo (MPRAGE) method in the following parameters: repetition time (TR)/echo time (TE)/inversion time (TI) / flip angle (FA) = 1900 ms/2.2 ms/900 ms/9°, acquisition matrix = 224 × 256 × 176, voxel size = 1 × 1 × 1 mm^3^. DTI parameters were: 12 non-linear directions (b-value = 1000 s/mm^2^) with 1 non-diffusion weighting acquisition, TR/TE = 6000 ms /85 ms, 30 axial slices, slice thickness = 5 mm, FOV = 256 × 256 mm^2^, acquisition matrix = 128×128, number of averages = 4.

### DTI Data Processing

DTI data were pre-processed and analyzed using the FMRIB's Diffusion Toolbox in FSL software (FMRIB Software Library, http://www.fmrib.ox.ac.uk/fsl) [30]. First, the original data was corrected for the effects of head movement and eddy currents using eddy correct command by applying an affine alignment of each diffusion-weighted image to the first b = 0 image. The non-brain tissue was removed by using the Brain Extraction Tool (BET) [31]. Then, the diffusion tensor elements were estimated and the corresponding FA and MD value of each voxel was calculated.

### TBSS Analysis

Tract-based spatial statistics of FA and MD images were adopted by using TBSS in FSL software [19]. FA images from all individuals were registered to Montreal Neurological Institute (MNI) 152 template using FNIRT, the non-linear registration tool in FSL. A mean FA image of all aligned FA images was calculated and then 'skeletonized' to create the study-specific mean FA skeleton, which represents the centers of all white matter tracts common for all subjects. Then, each individual’s FA and MD images were projected onto the skeleton. Voxel-wise statistics across subjects were calculated for each point on the skeleton.

### Statistical Analysis

Group statistical analysis was conducted only on voxels within the white-matter skeleton. Differences in FA and MD between aMCI-s, aMCI-m and NC were assessed by using analysis of covariance (ANCOVA), with age, gender and education level as covariates of no-interest. Nonparametric permutation test was used based on 5000 random permutations to generate a null distribution for each contrast. Threshold-free cluster enhancement (TFCE) was used in all comparisons to correct for multiple comparisons. The clusters with a TFCE-corrected P-value of less than 0.01 and a minimum cluster size of 50 contiguous voxels were reported. The John Hopkinson University (JHU) white-matter atlas [32] was applied to identify the names of WM bundles. Each cluster showed significant between-group difference was defined as a region of interest (ROI) and the confirmative analysis was then performed on each ROI to see the detailed relative patterns among the three groups. Finally, the partial correlation analyses were performed between mean WM index of each ROI and neuropsychological measures, including MMSE, MoCA, AVLT, BNT, TMT and CDT, with gender, age and education level as covariates. The threshold for the correlation analysis is corrected by using the Bonferroni corrections, i.e. (0.05/the number of ROIs).

## Results

### Demographic Information

As shown in Table 1, there were significant differences in age and education level among the three groups including NC, aMCI-s and aMCI-m, but no gender difference. There were also significant differences of AVLT, MMSE, MoCA (as well as CDR) among the three groups, however, these differences were mainly driven by the NC group while there were no significant difference between aMCI-s and aMCI-m. In particular, the significant group difference in BNT, TMT and CDT were found among the three groups, which then contributed to define the aMCI subtypes.

**Table 1:** Demographic characteristics of the three groups of participants including NC, aMCI-s and aMCI-m.

	NC	aMCI-s	aMCI-m	p value
Subjects (m/f)	23(10/13)	32(17/15)	23(13/10)	0.661*
Age	64.61±9.11	70.75±6.40	70.91±8.07	0.009^#^
Years of education	11.43±3.31	8.16±3.74	10.26±3.56	0.005^#^
AVLT	10.09±2.32	3.28±2.05	4.35±3.28	<0.001^#^
MMSE	28.43±1.50	24.09±3.30	24.48±3.93	<0.001^#^
MoCa	26.61±1.83	19.84±4.20	20.43±4.32	<0.001^#^
CDR	0±0	0.5±0	0.5±0	-
BNT	29.26±0.84	27.78±1.31	23.39±2.06	<0.001^#^
TMT	71.04±35.72	78.75±24.63	113.17±28.87	<0.001^#^
CDT	3±0	2.81±0.39	2.04±0.75	<0.001^#^

Values are means±SD. AVLT: the Auditory Verbal Learning Test (AVLT of Chinese version); MMSE: Mini-Mental State Examination; MoCA: the Montreal cognitive assessment; CDR: the Clinical Dementia Rating Scale; BNT: the Boston naming test; TMT: the trail making test; CDT: clock drawing test. The AVLT, MMSE, MoCA, CDT and BNT are based on number correct, CDR based on comprehensive rating scale, and TMT based on seconds.

* The p value was obtained using a Pearson x^2^ two-tailed test, with continuity correction for n < 5.

^#^ The p value was obtained using one-way ANOVA.

### DTI Results

**Fig 1:**
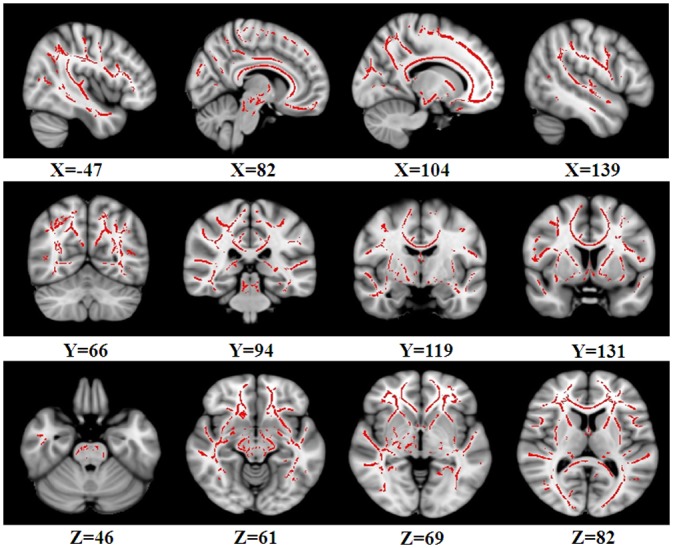
Regions showing significant FA differences among NC, aMCI-s and aMCI-m based on ANCOVA. The threshold was set at a TFCE-corrected P-value of less than 0.01 and a minimum cluster size of 50 contiguous voxels. Left is the left.

ANCOVA results showed that there were significant FA differences among the three groups including NC, aMCI-s and aMCI-m in the bilateral superior longitudinal fasciculus (SLF), left uncinate fasciculus (UF), left anterior thalamic radiation (ATR) and left inferior longitudinal fasciculus (ILF) (Fig 1 and Table 2). No cluster showed significant MD difference among the three groups.

**Table 2:** Regions showing significant FA differences among NC, aMCI-s and aMCI-m based on ANCOVA. The clusters with a TFCE-corrected P-value of less than 0.01 and a minimum cluster size of 50 contiguous voxels were reported.

Anatomical region	MNI			Voxels	p-value
	X	Y	Z		
Superior longitudinal fasciculus R	47	117	46	64041	<0.001
Uncinate fasciculus L	-82	131	61	368	<0.001
Superior longitudinal fasciculus L	-138	66	69	99	0.002
Anterior thalamic radiation L	-104	94	82	82	0.004
Inferior longitudinal fasciculus L	-144	119	53	64	0.003

**Fig 2:**
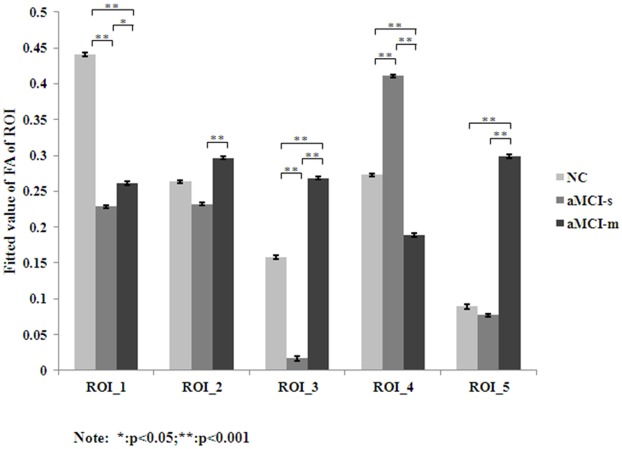
Paired-wise comparison of FA values in different ROIs. ROI were defined based on the activated clusters of ANCOVA (Table 1). * represents *p*<0.05; ** represents *p* < 0.001.

To further specify the variations in FA index, the activated clusters were defined as the regions of interest (ROI) and then post-hoc pair-wise comparisons were performed on mean FA values of each ROI (Fig 2). It was found that both aMCI-s and aMCI-m showed significant reduced FA than NC in the right SLF. It was also found that, in contrast to aMCI-m, aMCI-s showed significantly decreased FA in the left UF, left SLF and left ILF; while increased FA in left ATR.

### Correlations between FA and Neuropsychological Measurements

**Fig 3:**
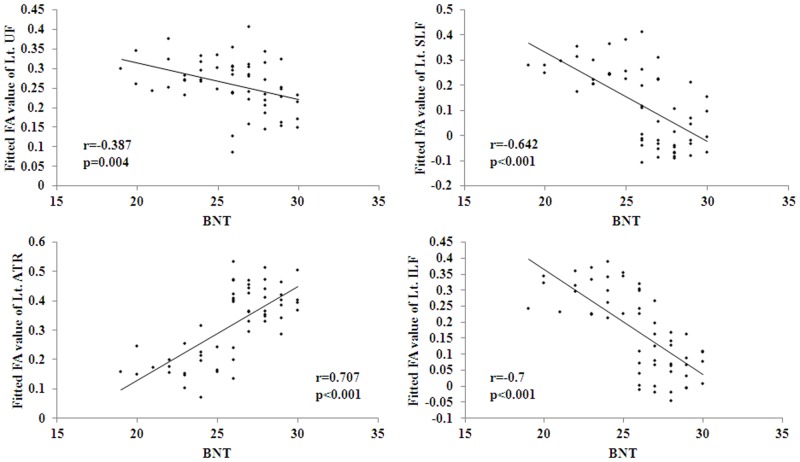
Significant correlations between FA values and BNT measures were found to be negative in Lt. UF, Lt. SLF and Lt. ILF, and positive in Lt. ATR. BNT: the Boston naming test.

**Fig 4:**
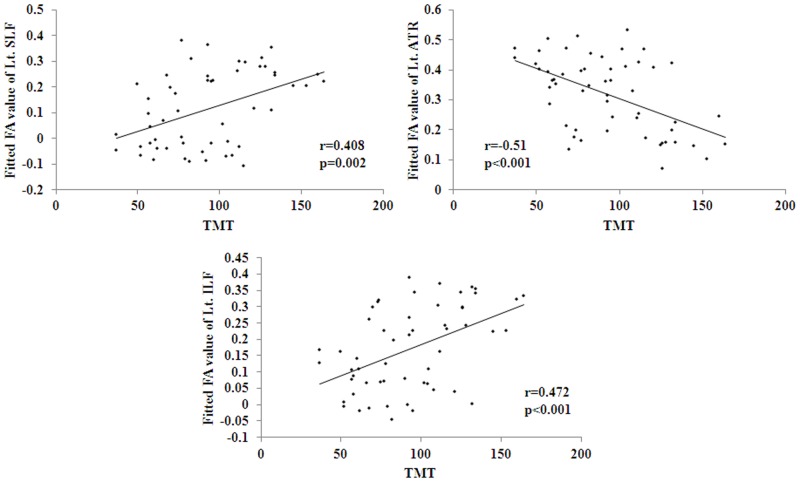
Significant correlations between FA values and TMT scales were found to be negative in Lt. ATR, and positive in Lt. SLF and Lt. ILF. TMT: the trail making test.

The partial correlation analyses with gender, age and education level as covariates were performed between mean FA of each ROI and neuropsychological measures including MMSE, MoCA, AVLT, BNT, TMT and CDT. It was found that BNT was negatively correlated with FA in the left UF, SLF and ILF, while positively correlated with FA in the left ATR (Fig 3). It was also detected that TMT was negatively correlated with FA in the left ATR, while positively correlated with FA in the left SLF and ILF (Fig 4). All the other correlations were not significant.

## Discussion

To our knowledge, this is the first study to examine the WM difference between the subtypes of aMCI. Our results showed that aMCI-s significantly differed from aMCI-m in some WM tracts, and the FA differences were associated with the cognitive deficits of patients. Particularly, some possible compensation effects were also identified. These results have added new evidence to the disconnection mechanism of aMCI [33–35], and further suggest that the two subtypes of aMCI had different structural connection damage, and FA may be a potential biomarker to differentiate aMCI-s from aMCI-m.

A larger cluster including the right SLF showed significant decreased FA in both aMCI-s and aMCI-m as compared to NC. This has added new evidences to the disconnection mechanism of MCI/AD. However, it was also found that a cluster in the left SLF indicated the different pattern that, in contrast to NC, aMCI-m showed increased FA while aMCI-s showed decreased FA. Specifically, FA values in the left SLF were significantly correlated with BNT and TMT, which suggested that the abnormality in the left SLF associated with the language and attention ability of aMCI patients. In particular, both the positive correlation between FA of the left SLF and TMT and the negative correlation between FA of the left SLF and BNT might indicate an effort of functional compensation (based on structural plasticity) in aMCI-m patients, although the language and attention ability of aMCI-m have been substantial damaged. This is totally congruent with the facts that SLF has a broad neuroanatomical extent, connecting the frontal, parietal, and temporal lobes [36], and plays an important role in higher-level cognitive functions including language [37–39]. However, we cannot exclude the possibility that white matter hyperintensity (WMH) in this area contribute for the abnormality in the left SLF, as the area is often affected by WMH.

This study found no significant alterations in the UF between NC and the two types of aMCI, although the UF was reported to be impaired in AD [40,41]. Particularly, FA values in the left UF might contribute to the differential diagnosis of the subtypes of aMCI, as there was significant FA difference between aMCI-s and aMCI-m. The UF is known for its involvement in human language functions [42–44], as it connects brain regions that have putative functions in language: the anterior temporal lobes and portions of the frontal lobes, both of which have been proposed to encode, store and retrieve semantic knowledge. In line with this, the significant increased FA in the UF in aMCI-m, in contrast to aMCI-s, might imply a kind of compensation mechanism, as evidenced by the negative correlation between FA in the UF and BNT.

The left ILF showed significant higher FA in aMCI-m as compared to aMCI-s and NC. It should be noted that the pattern in the left ILF was similar to that of the left UF. Actually, this might be explained by the facts that the ILF anteriorly joins the UF to relay information to the orbitofrontal brain [38], which means that the two fiber bundles linked and cooperated with each other to support some cognitive functions. In particular, the ILF is related to object cognition, verbal and visual memory as well as visuospatial cognition [8,45–47], and impairment of the ILF may induce thought disorders and cognitive impairment [48]. Thus, together with the significant correlations between FA in the left ILF and BNT as well as TMT, these results might also suggest a compensation mechanism in aMCI-m.

The ATR consists of fibers connecting mediodorsal and anterior thalamic nuclei to the prefrontal cortex and the anterior cingulate cortex. The left ATR in this study showed the different abnormal pattern from the other identified bundles. In contrast to NC, aMCI-m showed decreased FA while aMCI-s indicated increased FA. Further, the correlation between FA in the left ATR and BNT as well as TMT provided the possible explanation that the impairment of the left ATR contributes to the cognitive dysfunction in aMCI-m. Based on the identical logic to the aforementioned discussion, the relative higher FA in aMCI-s might indicate the compensation mechanism. These explanations were totally consist with the previous reports of the role of the ATR in cognition that the ATR is involved in working memory, executive function and planning complex behaviors [49,50]. This finding that the left ATR appears to be completely spared in aMCI-s but not in aMCI-m is also congruent with a previous work in CADASIL, which has shown that strategic lesions in this area can have consequences for impairments in multiple domains, including processing speed [51].

There were still some limitations in this study. First, the WMH within the white matter tracts assessed may substantially affect FA values. However, in this study, the WM lesions were not evaluated quantitatively and used as covariates to control for their effects when estimating the burden of FA on cognitive decline. Thus, it should be acknowledged that the findings are not necessarily independent of what is already known concerning the effects of WMH on cognitive performance. Second, given that the diffusion was 12 directions and 5mm thickness, which is not a optimized DTI protocol for high diffusion typically applied via TBSS. New experiment should be made to further validate the current findings with the optimized protocol. Third, this study was a cross-sectional design, and thus could not reveal the possible different progress characteristics of aMCI-s and aMCI-m. Furthermore, it was reported that, a novel technique, diffusion kurtosis imaging (DKI), enables characterization of non-Gaussian water diffusion behavior, and is considered to be more sensitive than DTI [52]. Given these advantages, we will also include DKI scan in the following studies. Finally, the current results were attained based on the Caucasian brain atlas. As the morphological characteristics of the western and Chinese populations are significantly different, in the future, the statistical Chinese brain atlas, such as Chinese2020 [53], may be used.

## Conclusion

Although there are common FA abnormalities between aMCI-s and aMCI-m in contrast to NC, the two types of aMCI own their own DTI characteristics separately. The findings in this study suggest that DTI might (in addition to traditional imaging and neuropsychological testing) serve as a sensitive technique for the differential diagnosis of aMCI-s and aMCI-m.
